# An Update on Nitric Oxide Production and Role Under Phosphorus Scarcity in Plants

**DOI:** 10.3389/fpls.2020.00413

**Published:** 2020-04-15

**Authors:** Andrea Galatro, Facundo Ramos-Artuso, Melisa Luquet, Agustina Buet, Marcela Simontacchi

**Affiliations:** ^1^Instituto de Fisiología Vegetal (INFIVE), CONICET-UNLP, La Plata, Argentina; ^2^Facultad de Ciencias Agrarias y Forestales, Universidad Nacional de La Plata, La Plata, Argentina

**Keywords:** abiotic stress, acid phosphatases, plant mineral nutrition, phosphate, reactive nitrogen species

## Abstract

Phosphate (P) is characterized by its low availability and restricted mobility in soils, and also by a high redistribution capacity inside plants. In order to maintain P homeostasis in nutrient restricted conditions, plants have developed mechanisms which enable P acquisition from the soil solution, and an efficient reutilization of P already present in plant cells. Nitric oxide (NO) is a bioactive molecule with a plethora of functions in plants. Its endogenous synthesis depends on internal and environmental factors, and is closely tied with nitrogen (N) metabolism. Furthermore, there is evidence demonstrating that N supply affects P homeostasis and that P deficiency impacts on N assimilation. This review will provide an overview on how NO levels *in planta* are affected by P deficiency, the interrelationship with N metabolism, and a summary of the current understanding about the influence of this reactive N species over the processes triggered by P starvation, which could modify P use efficiency.

## Introduction

It is not uncommon for plant roots to be exposed to temporary changes in local P availability. Considering the pivotal roles of this macronutrient in energy dynamics and metabolic regulation, P fluxes coordinately adjust to balance growth and development at the level of the whole plant. In the same way as it occurs with other mineral nutrients, both local signals acting on the cellular level, and long-distance or systemic signaling pathways, communicating internal nutrient status across different tissues and plant organs, must act coordinately to improve nutrient acquisition and internal utilization (Giehl et al., 2009; Lin et al., 2014; Ham et al., 2018; Wang et al., 2018; Ueda and Yanagisawa, 2019). The signaling compounds, such as NO and hormones, are involved in regulatory pathways when availability of nutrients is scarce (Giehl et al., 2009; Lei et al., 2011). In the case of P starvation, other signaling compounds come into play, including P itself, inositol polyphosphate, miRNAs, photosynthates, and calcium (Ruffel, 2018). Recently, a red-light signaling in the regulation of nutrient uptake and use was suggested, as it was found that expression levels of P starvation-responsive genes in Arabidopsis were modulated by PIF4/PIF5 and HY5 transcription factors, which activity is under the control of phytochromes (Sakuraba et al., 2018).

Reactive oxygen species (ROS) and NO have been recognized as early components in several mineral nutrient signaling events (Baxter et al., 2014; Kolbert, 2016). Even though there is a specific localization, timing and intensity of response depending on the depleted nutrient, it has been proposed that ROS and NO may be frequent elements in plant signal transduction cascades in response to nutrient imbalance (Xu et al., 2010; Zhu et al., 2019).

Sensing, signaling and the elaboration of acclimation responses will determine plant survival and performance in conditions of spatial and temporal variability of soil nutrient concentrations. To that end, in this review, we will discuss the current state of knowledge regarding NO functions in plants specifically under P restriction. We will focus on NO levels (and sources) in plants suffering from P deficiency, and its influence over the processes triggered by P starvation, which could modify P acquisition and use efficiency.

## Phosphate in Soil and Plants

Plants are often exposed to growth-limiting levels of P during their life cycle. As this is also true in crops, in order to maintain yields in low P soils, chemical P fertilizers obtained from mineral deposits are applied in each crop cycle (Baker et al., 2015). Each year around 148 million tonnes of phosphoric rock are mined, and 90% of that is used for food production (Cordell et al., 2009). A fraction of the applied fertilizer is lost to run-off, consequently reaching seas and lakes and resulting in eutrophication processes (Childers et al., 2011). Thus, current agriculture generates massive P mobilization from mineral deposits to water bodies: a one-way flux of a resource considered “non-renewable” (Cordell et al., 2009; Elser and Bennett, 2011). Throughout the whole process of P extraction and use, two main critical points can be identified: depletion of P reserves from mines, affecting future global food production’s sustainability, and eutrophication of water reserves, recently reviewed by Schoumans et al. (2014). It is clear that the understanding of diverse processes involved in soil-plant P dynamics constitutes a key point, to face not only an agronomic problem, but also worldwide environmental and economic ones, with a direct impact on water-food-energy security (Jarvie et al., 2015).

**FIGURE 1:**
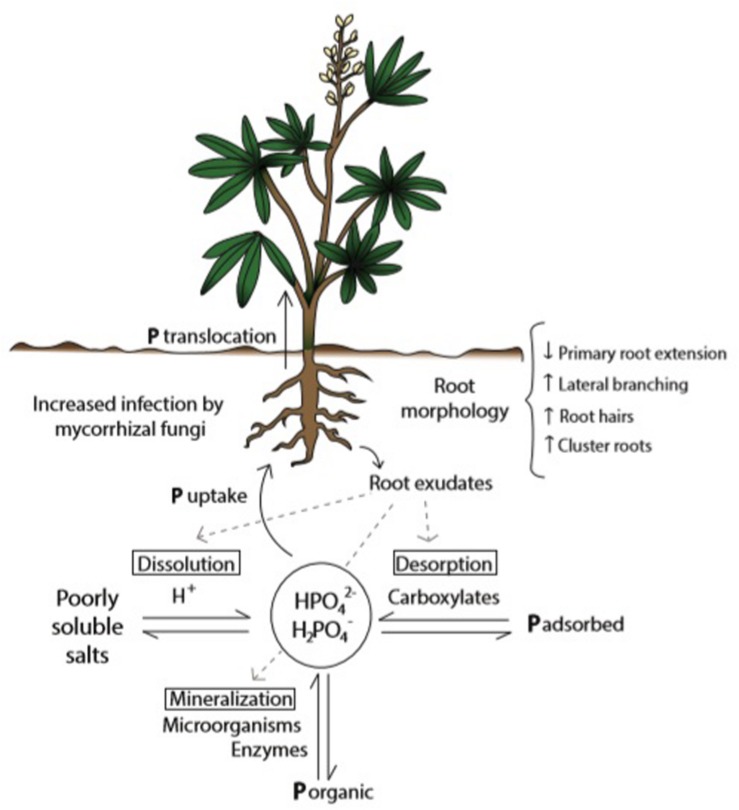
Plant’s strategies to improve P availability and uptake from soil solution in conditions of P scarcity. Acclimation responses tend to increase soil exploration (through morphological changes in roots), P desorption, dissolution, and interaction with microorganisms, as well as P internal remobilization (reallocation and interaction between P pools). P from the soil solution is taken up by the roots in the form of HPO_4_^2–^ and H_2_PO_4_^–^. Figure shows the dynamic equilibrium between available and not available P forms in soil, influenced by root exudations.

The fact that P in soil can be found in a variety of chemical forms – as organic compounds, in the mineral pool as poorly soluble salts, and adsorbed to particle surfaces – turns this nutrient into one of the least available for plant nutrition in the rhizosphere (Raghothama and Karthikeyan, 2005; McLaughlin et al., 2011; Shen et al., 2011). P from organic sources needs to be released through mineralization processes carried out by soil microorganisms and enzymes present in root exudates. Roots are able to uptake P from the soil solution in the form of H_2_PO_4_^–^ and HPO_4_^2–^, and whereas the dynamic balance of available and non-available P is determined by diverse soil and climate conditions (Marschner, 2011), it is also modified by plant roots (Wang et al., 2015), microorganisms (Khan et al., 2016), and other physiological traits (Pang et al., 2018). Therefore in this complex matrix only a small portion of total P in soil is available for plants (Pierre and Parker, 1927). As a consequence only 10–20% of P-containing fertilizers applied are available in the short term (reviewed by Chien et al., 2012). Plants with limited P supply induce changes in pH, organic acids (carboxylates) concentration, and activity of enzymes in the rhizosphere (Hallama et al., 2019), improving P solubility and availability (Figure 1).

Phosphorus is easily remobilized internally, and this is of great agronomic importance. According to some authors, P use-efficiency in crops is determined not only by uptake efficiency but also other factors including utilization inside the plant, once the P is taken up, and the production of economically relevant plant tissues per unit of incorporated P (Schröder et al., 2011). P remobilization increases under external P restriction or during senescence. Remobilization occurs, in general, from leaves but also from proteoid roots as is the case of the harsh hakea where ∼85% of P can be reallocated (Shane et al., 2014). Vacuoles are the primary intracellular compartments for inorganic P (Pi) storage, with active participation in remobilization (Yang et al., 2017). In vacuoles at pH 5.0, monoanion H_2_PO_4_^–^ predominates as the Pi species occurring in the efflux with a concentration in the millimolar range (Pratt et al., 2009). Using *in vivo* ^31^P-NMR, which allows for the discrimination between cytosolic-Pi and organelles-Pi pools, it has been found that, following the onset of P starvation, the Pi efflux from vacuoles is insufficient to compensate for a rapid decrease in the cytosolic Pi concentration. The sudden drop of cytosolic-Pi could be the first endogenous consequence to P starvation, triggering a signal transduction pathway which activates the P starvation rescue metabolism (Pratt et al., 2009). In addition to vacuole stock, when plants are exposed to P restriction, cell walls, membranes, RNA, and available organic compounds containing P, become important sources for the delivery of P to the actively growing tissues.

To better understand P fluxes in order to improve P use-efficiency by plants, a holistic interpretation of agroecosystems needs to be developed. This would identify the impacts of anthropic intervention through fertilization and diverse agricultural practices, soil P dynamics in connection with climate and microbiota, and plant physiological traits which modulate P acquisition and resulting internal reutilization (Macintosh et al., 2019).

Investigation of plant traits associated with agricultural P management is on the rise. New approaches are emerging such as the global-scale ecosystem services connected to soil fertility management (Macintosh et al., 2019) which is intended to overcome traditional agricultural perspectives, mainly focused on crop yield and short-term economic profits. In close connection with a global focus, P dynamic should also be studied at plant cellular and physiological levels. In this context, NO has influence on mechanisms which could increase soil exploration (Wu et al., 2014) and P availability (Ramos-Artuso et al., 2018), as well as mechanisms which could improve internal reutilization (Zhu et al., 2017). Thus, NO could be a key component in agro-ecosystem P flux interpretation, through modulation of P dynamics on plant physiological and plant-soil relationship levels.

## Nitric Oxide in Soil and Plants

### Nitric Oxide Synthesis in Plant Cells

Nitric Oxide can be endogenously produced by plant cells, but it can also be incorporated from the environment where it is generated as a result of activity of soil microorganisms. NO is a direct intermediate of nitrification (biological oxidation of ammonium, NH_4_^+^, to nitrate, NO_3_^–^) and denitrification processes (reduction of NO_3_^–^, to nitrogen gas, N_2_) carried out by microorganisms (Payne, 1976; Skiba et al., 1993; Feelisch and Martin, 1995). Soils are an important source of NO, and environmental conditions such as the presence of inorganic fertilizers can affect NO emissions. In this context, questions arise as to whether increased NO fluxes derived from N fertilization may affect growth and development, influence plant nutrition, and also affect a range of other plant responses (Lamattina et al., 2003). On the other hand, associative or symbiotic relationships between roots and microorganisms may contribute to NO production, and could also influence NO synthesis on each other (Molina-Favero et al., 2007).

Regarding NO synthesis, in mammals, nitric oxide synthase (NOS) enzymes use L-arginine, O_2_, and NADPH, to produce NO (Stuehr, 1999). In plants, different enzymatic and non-enzymatic sources can contribute to the generation of NO (Moreau et al., 2010; Fröhlich and Durner, 2011; Gupta et al., 2011; Mur et al., 2013; Astier et al., 2017), which have been classified as either oxidative or reductive pathways depending on the substrate involved (Gupta et al., 2011). NO production associated with NR, plasma membrane-associated nitrite:NO reductase (NiNOR) and other molibdo-enzymes (such as xanthine oxidoreductase, XOR), in addition to mitochondrial and chloroplastic electron transport chains, are all reductive pathways and depend on NO_2_^–^ as a primary substrate. Meanwhile, NO production from arginine, polyamines or hydroxylamine, belongs to the oxidative pathways.

Progress has recently been made concerning the two main sources of NO, arginine-dependent (known as NOS-like activity) and NR in plant tissues. Jeandroz et al. (2016) searched for the presence of transcripts encoding NOS proteins in over 1000 species of land plants and did not find typical NOS sequences. In photosynthetic organisms, only a few algae species contained NOS orthologs (Jeandroz et al., 2016; Santolini et al., 2017), such as the green alga *Ostreococcus tauri* (Foresi et al., 2010). However, the presence of proteins structurally unrelated to known NOS, or the cooperation between proteins or peptides, which combined can form a complex with similar NOS activity, cannot be discarded in higher plants (Fröhlich and Durner, 2011; Corpas and Barroso, 2017). In this scenario, NO production from NR seems to gain more relevance, considering the importance of NO_3_^–^ reduction and assimilation in plants. According to Chamizo-Ampudia et al. (2016), in *Chlamydomonas reinhardtii*, NR can supply electrons from NAD(P)H, through its diaphorase/dehydrogenase activity, to the molybdoenzyme NOFNiR (NO-forming nitrite reductase, also known as Amidoxime Reducing Component, ARC), which is in fact responsible for NO synthesis from NO_2_^–^, even in the presence of NO_3_^–^, condition under which NR is unable to do that. In addition, NR participates in the control of NO levels in cell by supplying electrons to the truncated hemoglobin THB1, which has dioxygenase activity (it can dioxygenate NO to produce NO_3_^–^). THB1 would then act by removing the very reactive NO and simultaneously inhibiting NR by uncoupling the electron transfer from NAD(P)H to NO_3_^–^ (redirecting the electrons from FAD to THB1). THB1 then plays a dual role in NO detoxification and in the modulation of NR activity (Sanz-Luque et al., 2015). Further research on the conditions that regulate NO or NO_2_^–^ production (such as the factors which favor the activity of NOFNiR and hemoglobins over NR) is required (Chamizo-Ampudia et al., 2017).

Over the last few years, research in the field of NO generation in plants has advanced. However, knowledge of the regulation of the multiple proposed pathways, especially under stress conditions, and in particular under mineral nutrient deprivation, is still limited. Different sources may come together, depending on stress conditions, substrate availability, species, and plant’s organs. Knowledge of the sources (and fates) of NO which operate under nutrient deficiency, in addition to the understanding of the specific roles of this molecule, are key tools to unraveling the mechanisms which trigger acclimation responses, where the modulation of endogenous levels of this molecule could be involved.

### Nitric Oxide Targets in Plants

**FIGURE 2:**
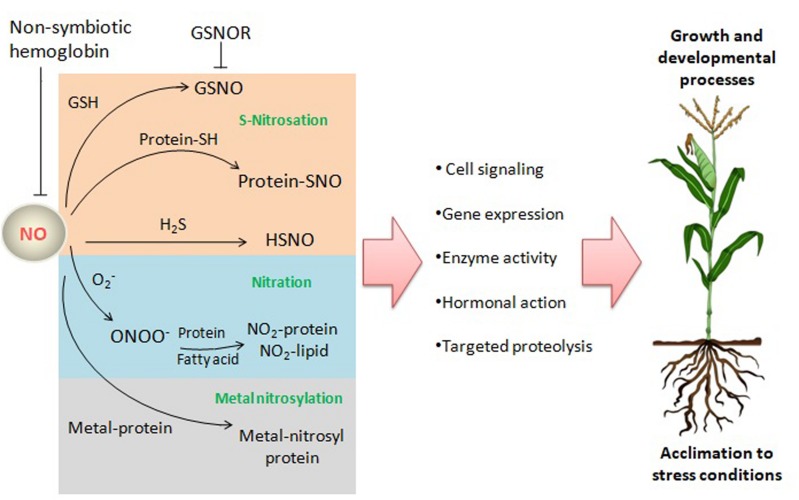
NO reactions and targets in plants. NO may exert its biological functions through interaction with ROS, the gasotransmitter H_2_S, proteins, and lipids leading to a broad range of biochemical events affecting signaling pathways and processes related to growth, development and acclimation to stress conditions. GSNO, S-nitrosoglutathione; GSNOR, GSNO reductase; O_2_^–^, superoxide anion; ONOO^–^, peroxynitrite; H_2_S, hydrogen sulfide; HSNO, thionitrous acid (the smallest S-nitrosothiol); Protein-SNO, nitrosated protein.

Nitric oxide may exert its biological functions through protein modifications, such as tyrosine nitration or S-nitrosation (also termed as S-nitrosylation), or through interaction with metalloproteins (metal-nitrosylation) (Figure 2), besides performing a broad spectrum of biochemical events through the interaction with hormones, and ROS among others (Gupta et al., 2020).

Although in animals the NO/cGMP-signaling (cyclic guanosine monophosphate) pathway has a main role in transmitting NO signal, plants have developed specific NO signaling different from other eukariotic lineages. According to recent studies, typical NO/cGMP signaling module is absent from representative plant species, while S-nitrosation has emerged as major NO-dependent signaling mechanisms in plants (Astier et al., 2019). Proteins and low-molecular-weight thiols can undergo S-nitrosation, where NO binds the thiol group of cysteinyl residues. S-nitrosation is a reversible post-translational modification by which NO can modulate protein activity.

S-nitrosoglutathione (GSNO) is the most abundant low-molecular-weight nitrosothiol and, in turn, it is considered to be a form of NO storage and long-distance transport (reviewed in Begara-Morales et al., 2019). NADPH-dependent thioredoxin reductase (NTR)/thioredoxin (Trx) system and GSNO reductase (GSNOR) control the extent of S-nitrosation through the regulation of GSNO levels and catalyzing denitrosation reactions (Begara-Morales and Loake, 2016).

Tyrosine nitration is mediated by reactive nitrogen species, such as ONOO^–^ and nitrogen dioxide (NO_2_), formed as secondary products of NO metabolism in the presence of oxidants, such as superoxide radicals (O_2_^–^), hydrogen peroxide (H_2_O_2_), and transition metal centers (Radi, 2004). In a relevant position, Tyr nitration can alter protein function and conformation, impose steric restrictions, and also inhibit Tyr phosphorylation. The result may be a loss-of-function (if a large fraction of protein is nitrated in specific critical tyrosines) or a gain-of-function (a small fraction of nitrated protein can elicit a substantive biological signal) (Radi, 2004). Evidence shows that protein Tyr nitration is an important NO-dependent signaling mechanism in plants, as well as nitration of fatty acids and ribonucleic acids (Arasimowicz-Jelonek and Floryszak-Wieczorek, 2019).

It is necessary to understand NO effects in the context of a complex network of molecules (Kolbert et al., 2019). The interaction of NO with other small signaling molecules, such as ROS and hydrogen sulfide (H_2_S), contributes to the regulation of growth, development, and of biotic and abiotic stresses responses (Singh et al., 2019; Figure 2). So far, there is insufficient knowledge of the specific crosstalk involving RNS, ROS and H_2_S under P-scarcity.

These mechanisms may account, in part, for the broad spectrum of NO actions in plants exposed to P imbalances, including posttranslational modification of proteins, enzymes, transporters and transcription factors, which may participate, in addition to hormones, in some of the physiological responses summarized here.

### The Levels of Nitric Oxide Are Influenced by P Deficiency

Wang et al. (2010) described a relationship between NO generation and P restriction in white lupin (*Lupinus albus*), where P deficiency enhanced NO production in primary and lateral root tips, with a bigger increase in juvenile and mature cluster roots (bottlebrush like structures). In searching for NO sources, root treatment with the mammalian NOS inhibitor (NG-nitro-L-arginine) and the XOR inhibitor (allopurinol) was found to reduce NO concentration in cluster roots, whereas with the NR inhibitor (tungstate), no effect of treatment was observed. Thus NOS-like activity and XOR may be two of the sources involved in NO production under P deficiency in white lupin.

In *Arabidopsis thaliana*, however, the NR double mutant (*nia1,2*) resulted in more sensitivity to P scarcity than WT plants, which indicated an impairment of the mechanisms involved in the acclimation to low P in these mutants. Moreover, P deprivation led to an increased NO production in roots from WT plants but not from *nia1,2*, which suggested a role for the NR pathway in NO production under P restriction conditions in Arabidopsis (Royo et al., 2015). In regards to soybean plants, P deprivation led to higher levels of NO in the leaves and an increase in NR activity as early as 24 h of P deprivation (Ramos-Artuso et al., 2019). Interestingly, the levels of total N, NO_3_^–^, NO_2_^–^, and the activities of other enzymes from N metabolism, were not affected. NR activity and NO generation may play a part in sensing P levels in cells triggering early metabolic responses to deal with P scarcity, as was suggested by the changes observed in the proteome in soybean leaves (Ramos-Artuso et al., 2019). Moreover, P deficiency induced an increase in NO level in rice roots (Zhu et al., 2016) where it was involved in cell-wall P reutilization, upstream of ethylene (Zhu et al., 2017).

Overall, NO seems to have a role in conditions of P scarcity in plants, not only in root response but also in shoot, where early changes are triggered as a consequence of P decrease. The two main ways of NO production in plants, NR and arginine-dependent, seem to be involved in the regulation of NO levels in the cell, but other potential sources (such as polyamines and other ways of NO_2_^–^ reduction) cannot be ruled out, and may also contribute to NO synthesis in conditions of P restriction. Future research will clarify this variable scenario dependent on multiple factors, including plant species, organ, source of N, and the level of P in the cell.

### Phosphorus Deficiency Affects N Metabolism and Impacts on NO Levels: Potential Regulatory Implications

It is known that P deprivation leads to alterations in N assimilation (Rabe and Lovatt, 1986; Rufty et al., 1993), but how these changes in N metabolism modify NO levels in cell and affect signaling events, is still unknown. Recent studies of P deficiency in plants have shown an increase in NO levels and suggest this molecule takes part in some responses related to acclimation conditions. Taking into account this scenario, changes in NO levels may either result in or be the result of alteration in N metabolism under conditions of P scarcity since N assimilation and NO generation are strongly connected. As mentioned before NO_2_^–^ and arginine, both derived from N assimilation and metabolism, are substrates for NO synthesis, and both the amount and the form of N supply (NO_3_^–^ or NH_4_^+^) could affect NO generation (Buet et al., 2019). Zhu et al. (2016) observed an increased NO content in roots from two rice cultivars under P-deficient conditions, where the feeding with NH_4_^+^ significantly increased the NO level as compared with NO_3_^–^ treatment. The effect of NH_4_^+^ over the NO levels was also observed in Arabidopsis plants with Fe deficiency (Zhu et al., 2019). In the case of soybean plants fed with NH_4_^+^, N assimilation occurs mainly in the roots, where the incorporation into amino acids is faster and stronger than with NO_3_^–^ as N source. This suggests a protective role, to avoid accumulation of high levels of NH_4_^+^, which can produce deleterious effects on cellular functions (Oliveira et al., 2013). In P-deficient leaves, it has been proposed that arginine biosynthesis may act as a protective mechanism for NH_4_^+^ detoxification (Rabe and Lovatt, 1986; Rufty et al., 1993). This highlights the need for an evaluation of arginine and polyamines levels in roots, as potential sources of NO in conditions of P deficiency.

In soybean (*Glycine max*), long-term P deprivation (20 days) led to several changes in N assimilation: rates of ^15^NO_3_^–^ uptake and net translocation of ^15^N from roots to shoots decreased, resulting in the alteration of NO_3_^–^ assimilation. Asparagine accumulated to high levels in stems and roots, and arginine accumulated to high levels in leaves was also observed (Rufty et al., 1993). Arginine levels increased in leaves of rough lemon (*Citrus limon*) and summer squash (*Cucurbita pepo*), when grown under P deficient conditions (Rabe and Lovatt, 1986). Nitrite levels increased in WT Arabidopsis roots after exposure of P restriction for 14-days (Royo et al., 2015) but not in soybean leaves after 24 h of P scarcity (Ramos-Artuso et al., 2019). However, in both plant species, NO increased and NR activity seemed to be involved in NO generation. If the amount of substrates for NO synthesis is affected under P deficiency, its availability may affect NO levels, but this factor is not the only one point of control for NO generation.

During P deficiency, the alteration of nutrient transport may be a non-specific result of changes in energy (low ATP), rates of water flow (hydraulic conductance), and membrane permeability; but other feedback control factors could affect NO_3_^–^ uptake (Rufty et al., 1993). It is worth mentioning that in *C. reinhardtii*, NO inhibited the high-affinity uptake of NH_4_^+^ and NO_3_^–^/NO_2_^–^, as well as NR activity in a reversible form which may include post-translational regulation (Sanz-Luque et al., 2013). It has been extensively noted that NO affects NR activity, but the effect depends on several factors, such as the N source (e.g., NO_3_^–^ concentration in the growth medium) and the level of NO or GSNO reached in each system (Buet et al., 2019, and references therein). Frungillo et al. (2014) has proposed that NO regulates NO_3_^–^ assimilation pathways and also controls its bioavailability by modulating its own consumption in *A. thaliana*. The authors observed that high levels of NO and S-nitrosothiols induced a switch from high- to low-affinity NO_3_^–^ transport reducing NO_3_^–^ uptake. The authors also observed that genetically elevated levels of GSNO inhibited the activity of NR while reduced levels promoted its activity. Also the enzyme GSNO reductase (GSNOR1) was inhibited by S-nitrosation. As this enzyme catalyses the NADH-dependent reduction of GSNO (stable pool of NO) to oxidized glutathione and NH_4_^+^, its inhibition could prevent this GSNO degradation and amplify S-nitrosothiols signals (Frungillo et al., 2014). Further research would highlight whether this regulation of N assimilation could also operate in conditions of P scarcity, modulate NO levels and, in turn, P deficiency responses.

In P scarcity conditions, diverse factors such as changes in the availability of substrates (as NO_2_^–^ or/and arginine) and modulation in the activity of some enzymes, including NR, XOR or NOS-like, can contribute to modify NO levels *in planta*. Despite NO origin, it may regulate N assimilation and, as a result, its own levels to participate in signaling events to afford P deficiency.

## Plant’s Acclimation to P Restriction

Plants sense low P availability, leading to the activation of a complex signaling network, which runs morphological, metabolic and physiological modifications often with great variations among species or even cultivars. In general, main modifications allow plants to enhance soil exploration, and P availability in the soil solution, as well as to maintain P homeostasis at the whole-plant level.

In this section we will discuss the extent to which the presence of NO could affect key plant acclimation responses to P scarcity (Table 1).

**TABLE 1:** Summary of NO effects on some physiological responses observed under P starvation and the potential components involved.

**Plant species**	**Treatment**	**Physiological effect observed**	**Major components involved**	**References**
*Lupinus albus*	P-deficiency (0 P) + SNP (50 μM)	Increased number of cluster roots and first-order lateral roots	Interaction with auxins (?)	Wang et al., 2010
		Increased concentration of citrate in exudation from cluster roots	Activation of plasma membrane H^+^-ATPase (?) Citrate metabolism (?)	
	P-deficiency (0 P) + cPTIO (500 μM)	Decreased number of cluster roots	Expression of genes that regulate cell division and radial root patterning	Meng et al., 2012
*Arabidopsis thaliana*	P-deficiency (5 μM P) + SNP (10–100 μM)	Primary root growth inhibition	DELLA proteins stabilization in root tip nuclei	Wu et al., 2014
*Arabidopsis thaliana (nia1,2)*	P-deficiency (0 P) + GSNO (200 μM)	Improved plant growth and alternative respiration rate	Alternative oxidase pathway (AOX)	Royo et al., 2015
*Zea mays*	P-deficiency (0 P) + GSNO (100 μM)	Increased Pi uptake from diluted solutions	P transporters (?)	Ramos-Artuso et al., 2018
		Increased P release from organic compounds	Activity of root APases	
		Increased external medium acidification	H^+^-ATPase (?) Citrate metabolism (?)	
*Oryza sativa*	P-deficiency (0 P) + SNP (2.5 μM)	Increased P translocation from roots to shoots	Expression of a phosphate transporter gene (OsPT2)	Zhu et al., 2016, 2017
		Increased soluble P	Cell wall pectin content	

*SNP and GSNO are NO donors, cPTIO is an NO scavenger, *nia1,2* is the nitrate reductase double mutant, (?) stands for proposed or potential components involved. Physiological effect refers to that observed as compared to P-deficient treatment.*

### Root Morphology

#### Cluster Roots

As a response to P starvation, root architecture sustains modifications which shows a higher presence of roots in the topsoil horizon- usually the P-enriched fraction- (Peret et al., 2014; Del-Saz et al., 2018). These morphological responses probably contribute to an efficient acquisition of this nutrient (Aibara and Miwa, 2014).

One of the most remarkable adaptations for some species consists in the development of proteoid or cluster roots when they are growing in conditions of P scarcity. These roots expose an enhanced surface area and strongly acidify the rhizosphere. White lupin (*Lupinus albus*) is frequently used as a model plant to study the formation and function of cluster roots under P-deficient conditions (Cheng et al., 2011). Shoot P concentration, sucrose and hormones (cytokinins, ethylene, and auxins) have been identified as participants in cluster root development (Cheng et al., 2011; Meng et al., 2013; Müller et al., 2015). In addition, after 32 days of P restriction, cluster roots showed a massive change in gene transcription (Venuti et al., 2019). A relationship between NO and morphological changes in roots has been described in lupin plants under P restriction. Using the fluorescent probe DAF-FM DA, NO was found to increase in the pericycle, endodermis cells and rootlet primordia from plants exposed to P restriction during 20 days. These results, and the timing of NO accumulation, led the authors to suggest NO was involved in the initiation, development and emergence of rootlets in the P deficiency-induced cluster-root formation (Wang et al., 2010; Meng et al., 2012). In addition, treatments with NO donors SNP and GSNO induced changes in roots morphology (Wang et al., 2010; Meng et al., 2012).

#### Primary and Lateral Roots

In species with non-proteoid roots, the reshaping of the root system architecture under P-deficiency usually includes the development of lateral roots and the inhibition of primary root extension (Peret et al., 2014). A crosstalk between auxins (known as root growth regulators) and NO has been described for cucumber explants. In this system, NO accumulation was detected after exposure to indolacetic acid (IAA, an auxin), and treatment with NO donors SNP (10 μM) and S-nitroso-N-acetyl-penicillamine (SNAP 10 μM) induced the production of adventitious roots to the same extent as IAA (Pagnussat et al., 2002). In tomato and maize plants, treatment with auxins (1-naphthylacetic acid and IAA) or SNP (200 μM) induced similar lateral root formation (Correa-Aragunde et al., 2004; Zandonadi et al., 2010). Further research has led to suggest that NO is required for cell cycle progression and establishment of lateral root primordia in the pericycle as NO modulates cell cycle regulatory genes (Correa-Aragunde et al., 2006). However, the relationship between NO and auxins in the context of root development under P deprivation still needs addressing.

The effect of NO on primary roots was described for tomato growing under sufficient nutrient conditions, where treatment with SNP (200 μM) strongly reduced primary root length, whose effect was reversed in the presence of NO scavenger cPTIO (Correa-Aragunde et al., 2004). In Arabidopsis, the inhibitory effect of exogenous NO treatment (SNP 10–100 μM) was observed for both P-sufficient and P-deprived plants (Wu et al., 2014). Even though the use of cPTIO confirmed a direct action of NO, there remained doubt as only the Fe-containing NO donor (SNP) was used as exogenous NO source (Wu et al., 2014). In Arabidopsis, it was described that localized Fe accumulation in the root tip – rather than the reduction in P concentration- was responsible for the growth inhibition under P restriction (Ward et al., 2008). Growth arrest requires accumulation of transcription factor STOP1 in the nucleus. Under P-restriction, it has been proposed that Fe stimulates the accumulation of STOP1 in root nuclei which, in turn, activates the transcription of malate transporter gene ALMT1 (Godon et al., 2019). The use of other NO donors, and the measurement of NO levels in each system, will confirm the effect of NO on primary root morphology under P scarcity.

Other mechanisms involving GA and NO may play a part in primary root inhibition under P restriction. Knowing that P starvation decreases GA biosynthesis in Arabidopsis, and that the addition of exogenous GA acts as a positive regulator for primary root growth (Jiang et al., 2007; Wu et al., 2014), the interaction between NO and GA on primary root growth has been analyzed and showed that the treatments, both with NO donor SNP and with NO scavenger cPTIO, modulated the effect of P restriction on primary root growth. In assays using Arabidopsis mutants in DELLA proteins (negative regulators of GA signaling), it was found that exogenous NO (SNP 10 μM) stabilized these proteins in root tip nuclei. Thus NO inhibition of primary root growth involves, at least in part, the DELLA-degradation pathway (Wu et al., 2014).

#### Root Hairs

Enhanced root hair development is a typical adaptive plant response to P starvation, where increasing root surface area for nutrient uptake is mediated by ethylene (Zhang et al., 2003; Song et al., 2016). The description of NO participation as a regulator of root hair development has long been documented. Lettuce plants treated with SNP (10 μM) showed increased root hair number and elongation, and Arabidopsis root hair development was inhibited by cPTIO (0.5–1 mM). Moreover, NO has been proposed as a mediator of auxin action in root hair growth (Lombardo et al., 2006).

Other nutrient’s availability usually affects root hair development, as is the case of Mg deficiency (Liu et al., 2017). Arabidopsis plants exposed to Mg deficiency exhibited increased levels of NO as well as ethylene, and, interestingly, both species were reciprocally influenced and interactively regulated root hair morphogenesis (Liu et al., 2017). Recently, a scheme including auxins has been proposed for plants exposed to Mg deficiency. In Arabidopsis plants subjected to Mg deficiency, both ethylene and NO were required to regulate the rise of auxins in roots (Liu et al., 2018). A positive feedback loop involving auxins, ethylene and NO production under Mg deficiency was found. However, there remains a lack of information regarding the role of ethylene, NO and auxins interaction in root hair development during P scarcity. A similar interaction between NO and phytohormones occurring under P-restriction regulating root hair growth could be anticipated.

### P Transport

The upregulation of high-affinity P transporters is a common phenomenon in response to P restriction in order to coordinate nutrient acquisition and distribution (Nussaume et al., 2011). As expected when compared with P-sufficient plants, there was a significant increase in the uptake of Pi from a diluted solution in maize plants exposed for 6 days to P-restriction, and the presence of an NO donor (GSNO 100 μM) further increased the potential capability of roots to incorporate P (Ramos-Artuso et al., 2018). Regulatory pathways integrated by microRNA and ubiquitin/26S proteasome degradation have been proposed for the selective modulation of P transport activity in response to P levels (Lin et al., 2014; Ye et al., 2018). The degradation of P transporters in P-sufficient conditions occurs through ubiquitin-mediated pathways which are reduced under P deficiency, activating Pi uptake as well as root-to-shoot translocation (Wang et al., 2017; Yue et al., 2017). In this regard, NO has been reported to be involved in ubiquitin-targeted protein degradation events in plants and animals (Arnaud et al., 2006; Kapadia et al., 2009). There is no knowledge directly related to how NO may affect P transport in plants; however, the activity of P transporters is regulated at both transcriptional and post-transcriptional levels (Nussaume et al., 2011), remarkably these processes are affected by NO under abiotic stress (Umbreen et al., 2018; Kolbert et al., 2019).

As mentioned before, P is easily redistributed, especially in plants under P-restriction. This includes the internal redistribution of P pools, the release of P from vacuoles, cell walls, and phospholipids in membranes, while at the whole plant level, P is reallocated to the young actively growing tissues (Veneklaas et al., 2012; Baker et al., 2015; Yang et al., 2017). P-translocation and the interconversion of different pools are related to the up-regulation of P transporters and enzymes (RNAses and phosphatases, among others), which release Pi from a broad range of P monoesters.

P starvation induced a specific group of cell-wall localized and intracellular APases, the PAPs (Shane et al., 2014; Ramos-Artuso et al., 2019), and in soybean the expression of a particular PAP (PAP21) increased in roots and old leaves, playing an important role in the utilization and recycling of P from intracellular reserves under P-starving conditions (Li et al., 2016). The same authors found that the overexpression of *GmPAP21* enabled plants to achieve around 96% higher fresh weight under P restriction as compared with WT plants, pointing out the role of this enzyme in internal P use efficiency in plants. Results of assays on maize plants (*Zea mays*) exposed to an NO donor during P restriction suggested NO played a part in the enhanced activity of root APases (Ramos-Artuso et al., 2018). Short term P-deprivation experiments (48 h) showed that the increase in gene expression and in activity of PAP1 in roots from *Medicago falcata* were dependent on the presence of the ethylene hormone (Li et al., 2011). Also in Arabidopsis, ethylene positively regulates P starvation-induced gene expression and activity of APase (Lei et al., 2011). In addition, during senescence of petunia corolla, ethylene regulated P remobilization and the expression of a P transporter gene (*PhPT1*) (Chapin and Jones, 2009). An interplay between NO and ethylene occurs not only during plant growth and development, but also when the plant is exposed to abiotic stress (Kolbert et al., 2019), as it has been proposed for rice, where P-restriction led to a quick enhanced NO production followed by an ethylene peak (Zhu et al., 2017). As a result, pectin content increased allowing reutilization of P from the cell wall, and at the same time, the expression of a P transporter (*OsPT2*) was up-regulated to facilitate the translocation of P from root to shoot, improving growth under P restriction (Zhu et al., 2016, 2017). To our knowledge this is the only study over the effect of NO (applied as SNP), showing that NO increases the expression of a P transporter gene under P deficiency (Zhu et al., 2017).

Since P-restriction produces an increase in NO (Wang et al., 2010; Ramos-Artuso et al., 2019) and in ethylene levels (Borch et al., 1999; Zhu et al., 2016), both species could interact, and contribute to optimizing P availability and translocation inside plant, affecting P use efficiency.

### Changes in the Rhizosphere

As a large proportion of P in soil is precipitated, chelated with metals, or becomes a constituent of organic compounds, the exudation of organic acids, protons and enzymes by roots contributes to improving plant’s P availability (Gaume et al., 2001; Shen et al., 2006; Pang et al., 2015). The “strategy” used varies greatly depending on plant species, genotypes and soil conditions (Hallama et al., 2019).

The induced secretion of ribonucleases, nucleases, phosphodiesterases, and APases is involved in P release from soil-localized organic substrates, such as nucleic acids and their degradation products, making P available for root uptake (Plaxton and Tran, 2011). Organic acids released from roots in the form of anions also contribute to increase P availability since they dissolve precipitates and chelate metal cations, and also block binding sites on soil particles. Citrate, malate and succinate from leaves can be transported via the phloem and directed to roots for exudation (Alexova et al., 2017). Citrate is the major organic acid released, and is one of the most effective species for solubilization of sparingly soluble P forms in soils (Igamberdiev and Eprintsev, 2016). NO was found to affect organic acid metabolism in citrus plants (*Citrus grandis*), where citrate content in roots increased after treatment with SNP (10 and 500 μM), due to an alteration in transport from leaves, whereas malate content increased (after treatment with 500 μM SNP), probably due to changes in the activity of the enzymes responsible for its synthesis and degradation (Yang et al., 2012). However, the dose of SNP used was toxic since plant growth was inhibited. As it has been previously discussed, lupin plants develop cluster roots under P-deficient conditions, characterized by their capacity to exude huge amounts of organic acids with citrate as the main component. Incubation of cluster roots from P-deficient lupin plants in the presence of an NO donor (SNP 50 μM, during 24 h) stimulated even more citrate exudation from the root, while the presence of cPTIO (an NO scavenger) had an inhibitory effect (Wang et al., 2010). Considering the side effects of SNP (León and Costa-Broseta, 2019), it would be interesting to test the effect of NO on organic acids production and exudation when using other NO donors in addition to carefully designed experimental approaches (such as the use of appropriate controls and scavengers).

Plant plasma membrane H^+^-ATPases transport protons out of the cell, controlling cell’s membrane potential and enabling the major transport processes in the plant, such as root nutrient uptake (Duby and Boutry, 2009). Enhanced H^+^ release to the rhizosphere occurs as a response to P-shortage and plays an important role in the acclimation to P-deficiency (Shen et al., 2006). Decrease in rhizosphere pH helps to dissolve P from Ca, Al or Fe phosphates and to increase P availability in calcareous soils (Hallama et al., 2019). It has been shown that the activity of plasma membrane H^+^-ATPase increases in P-restricted proteoid roots from lupin plants (Yan et al., 2002), and the enzyme has been described as an important component in responses to P restriction in soybean roots, where pharmacological and genetic approaches showed a parallel between enzyme activity and P uptake (Shen et al., 2006). Humic substances are naturally present in soils and, in addition to affecting root morphology, they stimulate the activity of H^+^-ATPase (Canellas et al., 2002). Interestingly, a link was found between NO and the humic-acid stimulation of H^+^-ATPase. In maize roots, plasma membrane H^+^-ATPase activity increased in the presence of NO donor SNP (200 μM), and the stimulatory effects of humic acids were inhibited by the addition of NO scavenger PTIO (Zandonadi et al., 2010). Moreover, data supporting a role for NO in the expression and activity of H^+^-ATPase were described in other systems under study. Calluses from reed (*Phragmites communis* Trin.) exhibited enhanced H^+^-ATPase activity 48 h after incubation with SNP (200 μM), and a reduced activity after treatment with PTIO (Zhao et al., 2004). None of the previous studies regarding the influence of NO or humic acids on the activity of H^+^-ATPase were performed under P restriction. Recently, maize plants exposed 6 days to P deprivation, being simultaneously in the presence of an NO donor (GSNO 100 μM), were found to exhibit higher capacity of external medium acidification as compared with P-deprived plants (Ramos-Artuso et al., 2018). It is worth considering that NO can be generated non-enzymatically in the apoplast, and its synthesis is favored when pH is low (Stöhr and Ullrich, 2002; Bethke et al., 2004). Thus, a mechanism in which NO induces external medium acidification, which, in turn, enhances NO synthesis under P-restricted conditions, may be suggested. Further experiments are necessary to unravel the role of NO over H^+^-ATPase activity in plants under P-restriction.

Apoplast acidification, through the activity of plasma membrane H^+^-ATPase, is also influenced by auxins, and it is involved not only in nutrient uptake, but also in cell growth (Rayle and Cleland, 1992). Auxins also induce accumulation of NO in roots, as has been evaluated in Arabidopsis, soybean, tomato, and cucumber (Pagnussat et al., 2002; Correa-Aragunde et al., 2004; Hu et al., 2005; Terrile et al., 2012). It is therefore possible to speculate an interplay between NO and auxins in the modulation of H^+^-ATPase activity under P scarcity.

### Symbiotic Associations

The broad range of adaptive P stress responses includes the establishment of symbiotic association with AM fungi (Mensah et al., 2015), through which plants can enhance their capacity to acquire P from soils by increasing root uptake volume, dissolving insoluble P, mineralizing organic P, and avoiding P slow diffusion from the soil solution to root surface (Zhang et al., 2014). AM symbiosis has been associated with changes in the amount of plant hormones in roots (cytokinins, auxins, ethylene, jasmonic acid, abscisic acid and strigolactones), and NO has recently been described as playing a role at the early stages of that interaction (reviewed in Martínez-Medina et al., 2019b). Five days after inoculation of *Medicago truncatula* with *Glomus mosseae*, the expression of the NR gene was significantly upregulated in roots. It was suggested that a diffusible fungal factor was perceived by the host tissues, since the same result was obtained when roots had been physically separated from *G. mosseae* hyphae by a semipermeable barrier (Weidmann et al., 2004). In search for a link between NR transcript accumulation and NO levels, roots of *M. truncatula* were exposed to fungal exudates obtained from germinating *Gigaspora margarita*, simulating the presymbiotic phase of the interaction (Calcagno et al., 2012). During the first minutes after exposure, increases in NO levels were found in the epidermal and cortical tissues of roots evaluated using DAF-2DA and confocal microscopy. Moreover, NR transcript level increased after 10 min of treatment, and AM-dependent NO accumulation was suppressed when NR activity was inhibited with tungstate (Calcagno et al., 2012).

Increased levels of NO, associated with AM-plant interaction, were also confirmed in: roots of *Trifolium repens* L. inoculated with *G. mosseae* (Zhang et al., 2013); olive roots in association with *Rhizophagus irregularis* (Espinosa et al., 2014); *P. trifoliata* seedlings colonized by *D. versiformis* (Zou et al., 2017); and tomato plants at the onset of the AM symbiosis with *R. irregularis* (Martínez-Medina et al., 2019a). Based on these results, Martínez-Medina et al. (2019b) has proposed a model for the establishment of mycorrhizal symbiosis which, during the pre-symbiotic stages, diffusible fungal signals are perceived by the plant, triggering a burst of NO linked with the activation of the symbiotic regulatory pathway. This partially suppresses the host immune responses and prepares the plant for fungal colonization. In later stages, the level of NO in root cells is controlled by the action of phytoglobins (Martínez-Medina et al., 2019b). However, experimental data regarding NO participation during mutualistic interaction in P-restricted conditions are not available. NO’s role in some key P-deficiency responses, on the one hand, and in the interaction with AMF, on the other, opens up interesting queries regarding whether integrated responses involving NO and soil microorganisms may occur under P scarcity.

## Concluding Remarks

To date there is little research on NO levels that have been conducted in plants exposed to P scarcity. NO increased after imposition of P-restriction treatments in lupin, Arabidopsis and rice roots, as well as in soybean leaves. NO seems to be implicated in acclimation responses to low P-availability, as it was described for citrate exudation, transcription and activity of a P transporter, P uptake from diluted solutions, external medium acidification and phosphatase activity, as well as modulation of some metabolic pathways. However, other functions of NO (for example, increasing H^+^-ATPase activity, role in symbiotic interaction, root hairs development, and lateral roots growth) remain to be studied in plants exposed to low P conditions.

Considering that a large amount of research has been developed using NO donors, caution should be taken regarding some observed side effects. In this regard, the use of other NO donors or scavengers in assays should be encouraged to strongly support some of the results. In addition, measurement of NO levels inside the plant as well as in different organs should be promoted to obtain a more integrated view.

Different mechanisms could be involved in NO synthesis during P scarcity but NR seems to have a critical role because of the close relationship between N metabolism and NO generation. Once NO concentration rises to a critical value, it may regulate its own specific level to exert a particular function. An interesting interaction of NO with hormones, such as ethylene, GA, and auxins during P deficiency may be proposed in some key acclimation responses. Moreover, future research in this field may confirm and show additional actors to this scenario. Overall, we encourage research in NO participation during P deficiency in order to find new tools to improve agricultural practices, avoiding fertilizers misuse and consequently, environmental damage.

## Author Contributions

AG, FR-A, AB, and MS contributed equally to conceive and write the manuscript. ML prepared the figures and contributed to the writing. All the authors approved it for publication.

## Conflict of Interest

The authors declare that the research was conducted in the absence of any commercial or financial relationships that could be construed as a potential conflict of interest.

## Abbreviations

AM: arbuscular mycorrhizal
APases: acid phosphatases
ARC: amidoxime reducing component
cPTIO: 2-(4-carboxyphenyl)-4,4,5,5-tetramethylimidazoline-1-oxyl-3-oxide
DAF-FM DA: 4-amino-5-methylamino-2′,7′-difluorofluorescein diacetate
GA: gibberellic acid
GSNO: S-nitrosoglutathione
IAA: indolacetic acid
N: nitrogen
NiNOR: nitrite:nitric oxide reductase
NO: nitric oxide
NOFNiR: NO-forming nitrite reductase
NOS: nitric oxide synthase
NR: nitrate reductase
P: phosphate
PAPs: purple acid phosphatases
Pi: inorganic phosphate
PTIO: 2-phenyl-4,4,5,5-tetramethylimidazoline-1-oxyl-3-oxide
ROS: reactive oxygen species
SNAP: S-nitroso -N-acetyl-penicillamine
SNP: sodium nitroprusside
WT: wild type
XOR: xanthine oxidoreductase.
